# Effect of Intravenous Chemotherapy Regimen on Globe Salvage Success Rates for Retinoblastoma Based on Disease Class—A Meta-Analysis

**DOI:** 10.3390/cancers13092216

**Published:** 2021-05-06

**Authors:** Anthony B. Daniels, Shriji N. Patel, Ronald W. Milam, Sahar Kohanim, Debra L. Friedman, Tatsuki Koyama

**Affiliations:** 1 Department of Ophthalmology and Visual Sciences, Vanderbilt University Medical Center, Nashville, TN 37232, USA; shriji.patel@vumc.org (S.N.P.); rmilam@ncretina.com (R.W.M.); sahar.kohanim@gmail.com (S.K.); 2 Department of Radiation Oncology, Vanderbilt University Medical Center, Nashville, TN 37232, USA; 3 Program in Cancer Biology, Vanderbilt University Medical Center, Nashville, TN 37232, USA; 4 Vanderbilt-Ingram Cancer Center, Vanderbilt University Medical Center, Nashville, TN 37232, USA; tatsuki.koyama@vumc.org; 5 Department of Pediatrics, Vanderbilt University Medical Center, Nashville, TN 37232, USA; debra.l.friedman@vumc.org; 6 Department of Biostatistics, Vanderbilt University Medical Center, Nashville, TN 37232, USA

**Keywords:** retinoblastoma, intravenous chemotherapy, intra-arterial chemotherapy, intravitreal chemotherapy, globe salvage rates, radiotherapy, Reese–Ellsworth classification, international classification of retinoblastoma

## Abstract

**Simple Summary:**

Intravenous chemoreduction (IVCRD) has been the standard of care for treatment of retinoblastoma (RB). Since survival rates exceed 95–98%, the goals of treatment have now shifted to emphasize eye salvage and vision preservation in addition to patient survival. Consequently, there has been a shift towards combining standard IVCRD with intravitreal chemotherapy or altogether replacing IVCRD with intra-arterial chemotherapy. As more data from intravitreal chemotherapy and intra-arterial chemotherapy are being published, there are claims of improved globe salvage rates resulting in more widespread use of these newer treatment modalities. However, there are no published randomized controlled trials comparing these to IVCRD head-to-head. To evaluate the relative efficacy of these new therapies, it is critical to determine the true success rates of IVCRD regimens alone. Therefore, it is both timely and essential to determine the baseline IVCRD success rates so that an evidence-based assessment of new and emerging therapies can be determined.

**Abstract:**

To evaluate the relative efficacy of novel retinoblastoma treatments, eye classification-specific success rates for current standard-of-care intravenous chemotherapy regimens must be known. This meta-analysis included studies if: (1) patients received intravenous chemotherapy for retinoblastoma, (2) globe salvage data was reported, (3) only *intravenous* chemoreduction (with/without local consolidation) was used. The outcome measure was globe salvage success without need for salvage radiotherapy, subdivided by disease classification and chemotherapy regimen. Data from 27 studies (1483 eyes) were pooled. By Reese–Ellsworth classification, globe salvage rates were 85% (95%CI:73–92%) for Group I, 78% (95%CI:70–85%) for Group II, 68% (95%CI:56–78%) for Group III, 47% (95%CI:34–60%) for Group IV, and 35% (95%CI:26–45%) for Group V (Va: 35% [95%CI:21–54%]; Vb: 42% [95%CI:29–56%]; those without sub-classification: 31% [95%CI:19–47%]). By International Classification, globe salvage rates were 93% (95%CI:80–97%) for Group A, 83% (95%CI:73–89%) for Group B, 73% (95%CI:54–86%) for Group C, 40% (95%CI:31–51%) for Group D, and 19% (95%CI:5–50%) for Group E. Standard carboplatin-etoposide-vincristine out-performed two-drug regimens (odds ratio (OR) = 1.9 (95%CI:1.3–3.0) for Groups I-IV and OR = 2.1 (95%CI:1.3–3.4) for Group V; *p* = 0.002 for each). For eyes with diffuse vitreous seeds (Vb), an enhanced regimen out-performed standard chemotherapy (OR = 2.4 [95%CI:1.3–4.7]; *p* = 0.004). In conclusion, two-drug regimens were less effective for all eyes, whereas enhanced regimens were more effective for eyes with vitreous seeds. Novel therapies can now be compared to these baseline globe salvage rates.

## 1. Introduction

Retinoblastoma is the most common primary intraocular malignancy in children [1,2]. In the 1990s, there was a shift in the paradigm for conservative management of retinoblastoma away from External Beam Radiotherapy (EBRT) toward intravenous (IV) chemoreduction (CRD) combined with local consolidation therapies (such as laser, cryotherapy, or transpupillary thermotherapy) [3,4,5,6,7]. As the management of intraocular retinoblastoma with CRD has advanced, patient survival rates now exceed 95–98% in developed countries [1,8,9]. Although the focus continues to be on maximizing patient survival and safety of treatment, the emphasis is now also on optimizing eye (globe) salvage and vision. As a result, there has been a substantial reduction in the rate of enucleation over the past few decades [10,11,12,13], and the use of EBRT has sharply declined [8].

Although the standard retinoblastoma treatment regimen with CRD consists of six cycles of Vincristine (V), Etoposide (E) and Carboplatin (C), various other regimens have been reported. These include reduced regimens consisting of only two drugs to minimize side effects, and enhanced regimens (with additional [and sometimes more toxic] second-line agents, or additional cycles of therapy beyond the standard 6 cycles) to attempt to improve globe salvage [7]. Furthermore, given the diversity of clinical presentations (number of tumors, eyes, presence of seeds), the management of retinoblastoma can be extremely complex, and thus it is virtually impossible to establish a standard set of rules for the choice of treatment. In general, small tumors have been treated with local treatments, while larger tumors have been traditionally treated with CRD, augmented by local consolidation measures.

Recently there has been an increasing shift to either replacing CRD with intra-arterial chemotherapy (IAC; with chemotherapy injected endovascularly via microcatheter into the ophthalmic artery of the eye) or combining “standard” IV CRD with adjuvant treatments such as direct intraocular injection of chemotherapy (“intravitreal chemotherapy”). There is mounting evidence that additional eyes can be salvaged with IAC or with adjuvant intravitreal chemotherapy compared to IV CRD alone [14,15,16,17,18,19,20,21,22,23,24]. However, there are no published randomized controlled trials comparing IAC to IV CRD head-to-head.

### Rationale and Objectives

In order to scientifically evaluate the relative efficacy of these new treatment modalities, it is critical to determine the actual success rates of current IV CRD treatment regimens, to establish a baseline against which to evaluate new, emerging, and future therapeutic options. The use of IAC and intravitreal chemotherapy is becoming more widespread, and in some centers, has led to the complete or near-complete replacement of standard IV CRD [18]. As more results of experience with IAC and intravitreal chemotherapy are being published from various treatment centers, there are claims of improved globe salvage rates with these new therapies [25,26,27,28]. It is therefore very timely, and critical to interpreting the relative efficacy of new therapies, to determine the baseline rates of globe salvage with traditional CRD, to allow a sounder, evidence-based assessment of claims of improved efficacy with these newer treatments.

This goal of determining a baseline globe salvage rate for IV CRD is complicated by two main factors. First, both treatment approach and globe salvage success are highly variable between affected eyes, based on location and size of tumor(s), or the presence of difficult-to-treat subretinal or vitreous tumor seeds. Thus, it is necessary to stratify tumor burden according to one of the established retinoblastoma classification systems to compare between studies. Second, while six cycles of IV VEC is often referred to as the “standard” regimen for intraocular retinoblastoma, there are actually a multitude of regimens that have been reported by various major retinoblastoma treatment centers [4,5,6,11,19,29,30,31,32,33,34,35,36,37,38,39]. Therefore, we performed a meta-analysis of the published literature of IV CRD for retinoblastoma in children, in order to systematically evaluate globe salvage success rates according to each of the retinoblastoma classification systems (Reese–Ellsworth [R-E] and International Classification of Retinoblastoma [ICRB]/International Intraocular Retinoblastoma Classification [IIRC]) and to allow comparison of different chemotherapy regimens.

## 2. Materials and Methods

### 2.1. Eligibility Criteria

We conducted a literature review and meta-analysis to assess for reported globe salvage success rates for the treatment of intraocular retinoblastoma, according to eye classification, for various IV chemotherapy treatment regimens. Search terms included “retinoblastoma” and either “chemotherapy” or “chemoreduction”. For inclusion in the meta-analysis, studies must have only included reports of IV chemotherapy for the treatment of intraocular retinoblastoma in human patients, with or without adjuvant focal consolidation (laser, cryotherapy, transpupillary thermotherapy, or plaque brachytherapy). In vitro and animal studies, studies unrelated to the treatment of retinoblastoma, or studies using a treatment modality other than IV chemotherapy were excluded. Case reports, small case series and editorials were also excluded. Studies that included IV CRD in conjunction with IAC, or which reported on IV CRD in conjunction with intravitreal chemotherapy were excluded. Of the remaining, studies were included only if the exact number of eye treatment failures was clearly reported, or if the studies had individual patient level data where we could evaluate the number of treatment failures (treatment failure by our criteria was defined as eyes requiring salvage EBRT or enucleation).

### 2.2. Search Methods

PubMed was searched for English language articles containing the words “retinoblastoma” and “chemotherapy” for all articles published prior to 1 January 2017, yielding 2710 papers. We selected 1 January 2017 as an end date, as intravitreal chemotherapy began to become widely used in conjunction with intravenous chemotherapy at many retinoblastoma treatment centers after this time. Three expert reviewers, a vitreoretinal specialist (SNP), an ocular oncologist (ABD) and a pediatric oncologist (DLF), reviewed all of the studies using a 2-step study selection process (title/abstract screening followed by full text evaluation) to determine which ones met inclusion criteria. Additionally, references in the identified studies were also screened for additional appropriate articles.

### 2.3. Study Selection

The studies which satisfied the inclusion/exclusion criteria were then evaluated for the type of IV chemotherapy regimen used, “standard” CRD (consisting of 4–6 cycles of VEC), two-drug regimens (VE, VC, or EC) and “enhanced” regimens (consisting of multiple additional cycles of VEC, or including a fourth chemotherapy agent, or using alternate, more toxic second-line chemotherapy agents). These studies were then evaluated independently by three data extractors (SNP, ABD, DLF) to determine the treatment success for each chemotherapy regimen used. A fourth reviewer (RWM) evaluated all extracted data for accuracy. Treatment success was defined as globe retention without the need for salvage EBRT or enucleation. Studies with planned EBRT were excluded from our analysis. Studies in which eye classification (RE or ICRB/IIRC) could not be determined were excluded, as were papers in which it was not possible to identify which eyes avoided both EBRT and enucleation (which would define the treatment as a failure by our criteria). Abstracts and unpublished studies were excluded. Duplicate papers reporting on previously published cohorts of patients were also excluded.

### 2.4. Data Collection

Three reviewers independently extracted data into a customized database. Extracted data for each study included title, authors, publication year, number of patients, number of eyes treated, IV chemotherapy regimen used (standard, two-drug or enhanced), number of eyes in each classification for both ICRB/IIRC and RE systems, and number of globe salvage successes and failures (by our criteria described above). There are two different versions of the International System (the ICRB and the IIRC). For the purposes of analyses, these were grouped. See the Discussion section for a full discussion of this point.

### 2.5. Data Synthesis and Analysis

For aggregate data, random-effects meta-analysis was conducted to estimate the globe salvage rate for each R-E eye group (I–V) and for each ICRB/IIRC eye group (A–E). Inverse-variance weighted point estimates and 95% confidence intervals (Clopper–Pearson) for success rates by classification group (R-E and/or ICRB/IIRC) were calculated. We also assessed different success rates based on chemotherapy regimen. Chi-square test for trend in proportions was conducted for the globe salvage rate for R-E classification and ICRB/IIRC classification. Where individual patient-level data were available, we analyzed the individual data in addition to the aggregate data. Only eyes treated with CRD were included in our analyses. Eyes treated primarily with enucleation were excluded. When data on both eyes were reported for a patient with bilateral disease who received CRD to treat both eyes, the more severe eye was chosen for this analysis, to avoid issues of inter-eye correlation in statistical analysis. A logistic regression model was fit with patient age, chemotherapy type (Reduced/Standard/Enhanced) and eye disease classification group as covariates. Treatment failure was defined as those eyes requiring either enucleation or salvage EBRT. To account for within-study correlation, the Generalized Estimating Equation (GEE) method with independent weight matrix was used. Data analysis was performed using the R software package (R: A language and environment for statistical computing. Version 3.4. R Core Team, R Foundation for Statistical Computing, Vienna, Austria).

In two parallel analyses, we examined globe salvage success as a function of disease classification based on the IV chemotherapy regimen used. Chemotherapy regimen was grouped as either standard, reduced, or enhanced (as defined above). Reduced intensity regimens were compared to standard CRD for both less advanced (R–E groups I–IV) eyes, as well as for more advanced (R–E group V) eyes. Enhanced regimens were compared to standard CRD for those eyes with the most difficult to treat disease, that is, R-E group Vb eyes with vitreous seeds, as we felt that these were the eyes where the added toxicity of enhanced regimens might be most-warranted, and where such a second-line regimen might be most strongly considered. The reasoning behind this is discussed more fully below in the Results and Discussion sections.

A second analysis was performed based on individual patient level data. Of all 93 studies that met our inclusion criteria, sufficient individual patient level data were reported in 14 studies [4,30,31,32,33,39,40,41,42,43,44,45,46,47]. Of these 14 studies, all used R-E classification except for one [43], which reported eyes using the ICRB classification system. As the other 13 studies stratified based on the R-E classification, this single study [43] was excluded from the individual patient-level analysis. Two additional studies [4,47] were removed for missing patient-age information, since we felt age was an important covariate. We therefore limited our multivariable analysis of individual patient level data to these 11 studies.

## 3. Results

### 3.1. Study Characteristics

**Figure 1:**
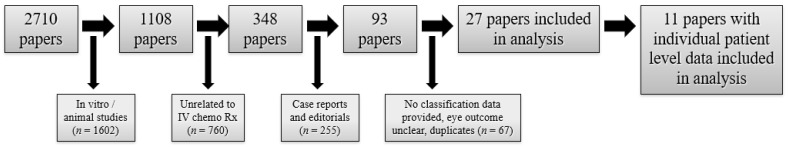
Study inclusion/exclusion algorithm. Studies were excluded if they involved in vitro or animals (*n* = 1602), used a treatment modality other than IV chemotherapy (*n* = 760), were small case series or editorials (*n* = 255) or had no disease classification data or unclear outcomes (*n* = 67).

The literature search methodology above resulted in 2710 reports that were then screened further using our specific inclusion/exclusion criteria (Figure 1). Of these 2710 studies, 93 studies met the initial inclusion criteria, reporting on the treatment of intraocular retinoblastoma in human patients with IV CRD therapy. Of these 93 studies, 27 [4,5,29,30,31,32,33,34,35,36,37,38,39,40,41,42,43,44,45,46,47,48,49,50,51,52,53] included sufficient data on eye classification and outcomes to be included in this meta-analysis (Figure 1 and Table 1). The median number of eyes included in these studies was 43 (range 10–249 eyes). Eight studies [4,31,32,43,44,45,47,48] used enhanced CRD (either higher doses, additional drugs, or a greater number of treatment cycles), 12 studies [5,30,33,34,35,36,37,38,40,41,42,49] used two-drug regimens, and 12 studies [5,29,32,33,39,44,45,46,50,51,52,53] used standard CRD with 4–6 cycles of standard-dose VEC. In total, 23 studies [4,5,29,30,32,33,34,35,36,37,38,39,40,41,42,43,44,45,47,48,49,52,53] reported R-E tumor classifications, while 10 studies [31,38,43,46,47,48,49,50,51,52] reported ICRB/IIRC tumor classifications, and 6 studies [38,43,47,48,49,52] classified eyes according to both the R-E and ICRB/IIRC classification systems. Two studies [39,43] were restricted to only reporting on R-E Group V eyes, while 1 study [44] was limited to only reporting R-E Group IV or V eyes. Similarly, 2 studies [50,51] were limited to only reporting ICRB Group D eyes. Thus, 5 studies reported on success rates for only more advanced eyes [39,43,44,50,51] (Table 1).

Across all studies, we collected data on a total of 1483 eyes. Of these, 1308 eyes were classified according to the R–E classification, with 158 Group I, 214 Group II, 232 Group III, 122 Group IV and 582 Group V eyes. In total, 764 eyes were classified according to the ICRB/IIRC system, with 59 Group A, 241 Group B, 74 Group C, 348 Group D, and 42 Group E eyes. Therefore, in the reported literature, there is an overrepresentation of published IV CRD treatment outcomes for more advanced eyes (R-E Group V and ICRB/IIRC Group D eyes) (Table 1).

### 3.2. Globe Salvage Rates among All Retinoblastoma Eyes by R-E and ICRB/IIRC Classification

**Figure 2:**
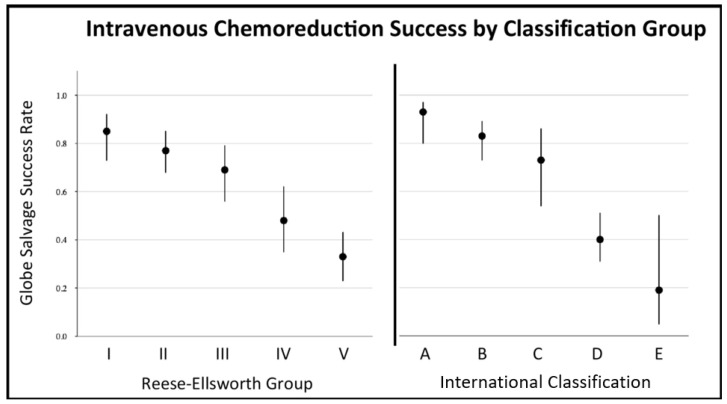
Globe salvage success rates by Reese–Ellsworth (left) and International Classification of Retinoblastoma/International Intraocular Retinoblastoma Classification (right), showing weighted point estimates with 95% confidence intervals. This trend was highly significant for both Reese–Ellsworth classification (*p* < 0.001) and ICRB/IIRC (*p* = 0.001).

Globe salvage was defined as those eyes that did not require either enucleation or treatment with EBRT for salvage. Among all included studies, globe salvage rates varied based on severity of the disease, with both R-E and ICRB/IIRC classification systems predicting globe salvage rates. Regardless of classification system used, eyes classified as having worse disease reliably demonstrated lower successful globe salvage rates, compared to eyes classified as having less severe disease (Figure 2). Weighted estimates (with 95% confidence intervals [95%CI]) of globe salvage rates for R-E classification tumors were 85% (95%CI:73–92%) for Group I, 78% (95%CI:70–85%) for Group II, 68% (95%CI:56–78%) for Group III, 47% (95%CI:34–60%) for Group IV, and 35% (95%CI:26–45%) for Group V eyes (including 35% (95%CI:21–54%) for Va, 42% (95%CI:29–56%) for Vb, and 31% (95%CI:19–47%) for group V eyes without sub-classification). This trend was highly significant (*p* < 0.001). Using the ICRB/IIRC classification, weighted globe salvage rates were 93% (95%CI:80–97%) for Group A, 83% (95%CI:73–89%) for Group B, 73% (95%CI:54–86%) for Group C, 40% (95%CI:31–51%) for Group D, and 19% (95%CI 5–50%) for Group E eyes. Again, this trend was highly significant (*p* = 0.001) (Figure 2).

### 3.3. Globe Salvage Success Rates of Chemotherapy Regimens and the Effect of Disease Classification

We considered 4–6 cycles of standard dose VEC as the “standard” regimen. Across all 27 studies (*n* = 1483 eyes), a “standard regimen” was used in 45% (*n* = 668) of eyes. Two-drug regimens included only VE, VC, or EC, and these were used for 30% (*n* = 442) of eyes. Finally, 25% (*n* = 373) of eyes were treated with an “enhanced” regimen (>6 cycles of VEC, or higher-than-standard doses, or additional or second-line chemotherapy agents).

We compared two-drug regimens to standard regimens, not just for advanced (group V) eyes, but also for groups I–IV eyes, based on the clinical judgment that it would primarily be in patients with groups I–IV eyes that a reduced-intensity regimen might be considered. This is consistent with the Children’s Oncology Group study which examined a two-drug regimen for less advanced (ICRB group B) eyes [54]. We determined that analysis of enhanced regimens would be of most clinical utility in those eyes that are known to be hardest to treat, that is, R-E group Vb eyes with difficult-to-treat vitreous seeds. In these eyes, increasing the intensity of the IV CRD regimen may be a clinically relevant strategy, especially if weighing the need for an alternative treatment modality like IAC. Enhanced regimens were not considered in less advanced eye groups, as second-line chemotherapy agents in the treatment of less advanced disease would not be considered standard of care outside of a clinical trial. By pre-defining these study groups and limiting our analyses to those treatment decisions that would be of most utility clinically, we also avoided mining the data. Due to the relative paucity of studies that used a non-standard IV CRD regimen while simultaneously employing the ICRB/IIRC system, this analysis of the impact of IV chemotherapy regimen was not performed separately based on ICRB/IIRC classification.

**Figure 3:**
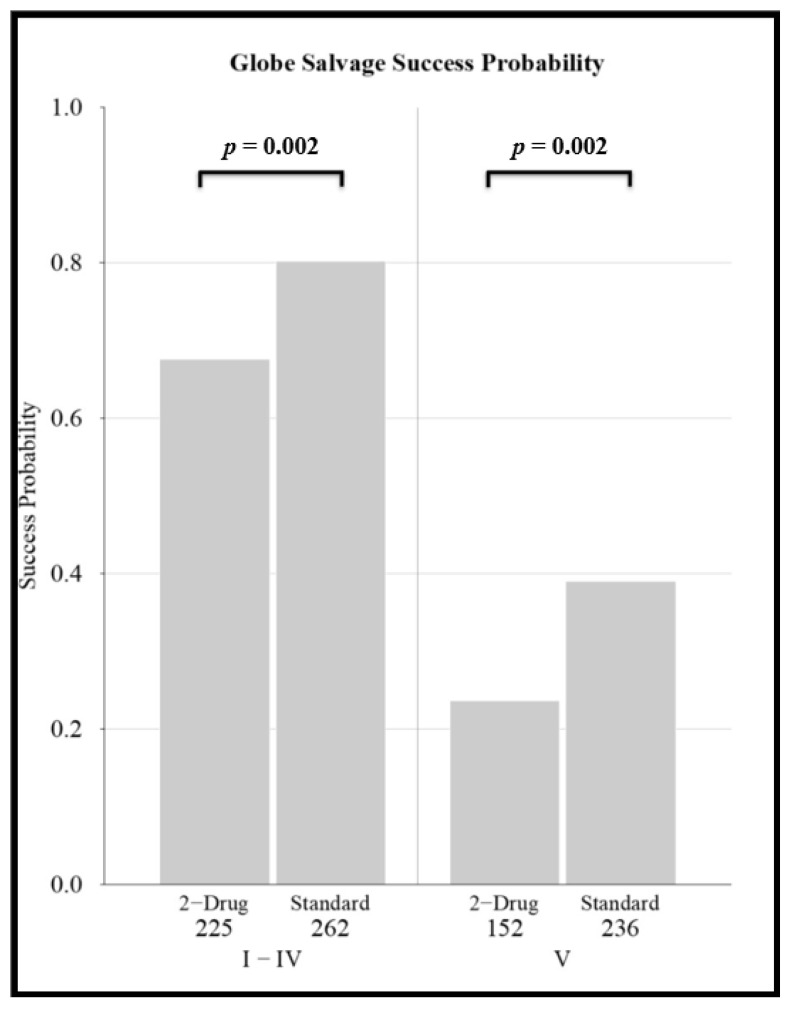
Comparison of globe salvage probability for “reduced” (2-drug regimen) vs. “standard” intravenous chemotherapy regimens for less advanced (Reese–Ellsworth groups I–IV) and more advanced (Reese–Ellsworth group V) eyes.

**Figure 4:**
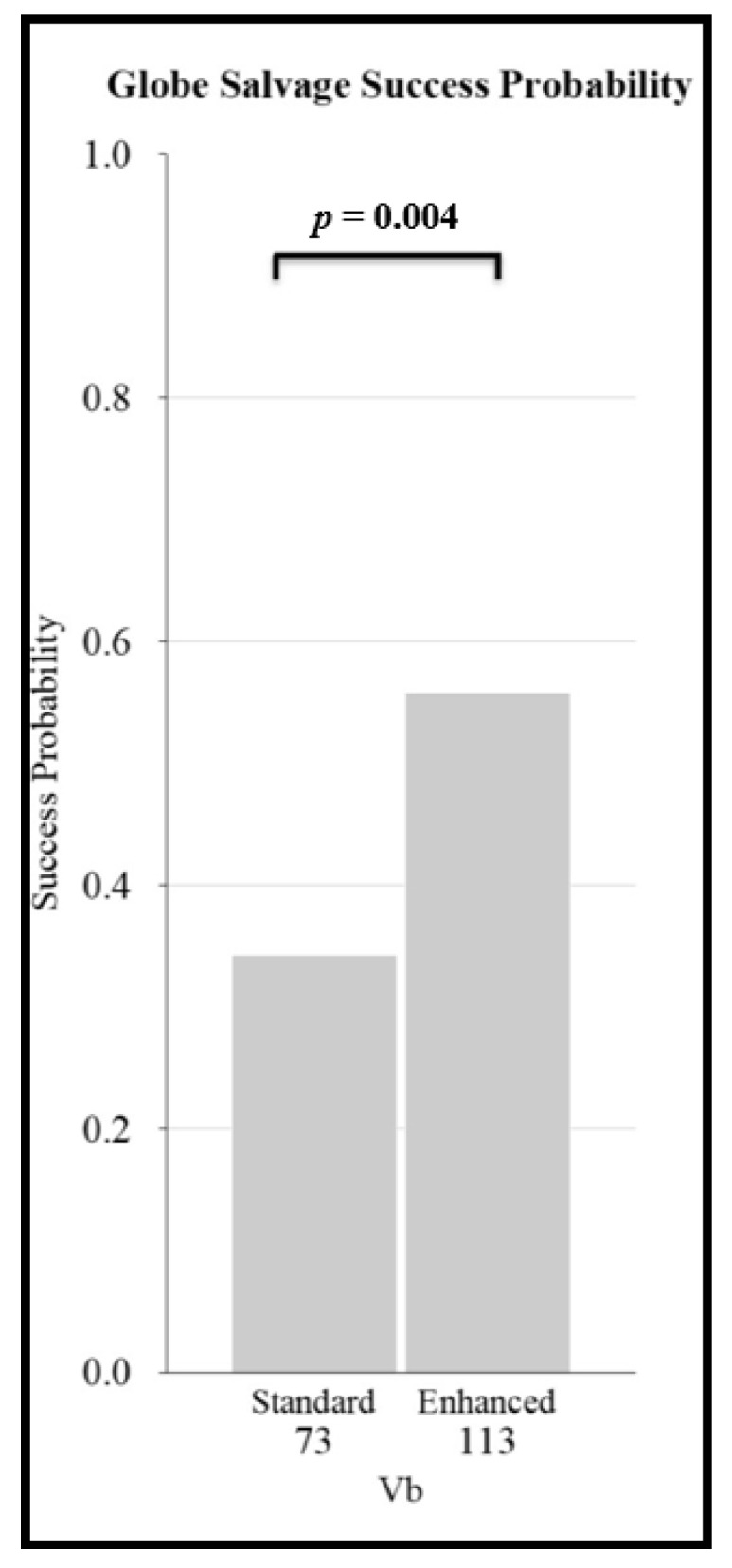
Comparison of globe salvage probability for “standard” vs. “enhanced” intravenous chemotherapy regimens for the most advanced eyes with vitreous seeds (Reese–Ellsworth group Vb).

For less advanced eyes (groups I-IV) the odds ratio for globe salvage was 1.9 (95%CI:1.9–3.0) for standard regimens vs. two-drug regimens (*p* = 0.002). For more advanced (group V) eyes, the odds ratio was 2.1 (95%CI:1.3–3.4) for standard regimens vs. two-drug regimens (*p* = 0.002) (Figure 3). For the most advanced eyes with vitreous seeds (group Vb), the odds ratio was 2.4 (95%CI:1.3–4.7) for enhanced regimens vs. standard regimens (*p* = 0.004) (Figure 4).

### 3.4. Individual Patient Level Data: Effect of Patient Age, Eye Classification, and Chemotherapy Regimen on Globe Salvage Success

Individual patient level data were available for analysis in 11 of the 27 included studies [33,34,35,36,37,38,40,41,42,44,45], with 6–25 eyes per study. The total aggregated sample size was 202 eyes. Regimens were grouped as described above. Among studies with individual patient level data, standard regimens were used in only 18% (*n* = 36) of eyes, two-drug regimens were used in 69% (*n* = 139) of eyes, and enhanced regimens were used in 13% (*n* = 27) of eyes.

On an individual patient level, older age and more advanced disease (Group V, as compared to groups I–IV) were independent predictors of worse prognosis for globe salvage, regardless of chemotherapy regimen used. The odds of successful globe salvage were 4.3 (95%CI:2.8–6.5) times higher for groups I–IV compared to group V. The odds of successful globe salvage decreased by almost half for every 10-month increase in age (Odds Ratio = 0.53 [95%CI:0.36–0.79]), independent of disease severity or treatment regimen.

## 4. Discussion

### 4.1. Summary of Evidence

Our analyses reconfirm that both the R-E and the ICRB/IIRC classification systems are predictive of chemoreduction success. Two-drug CRD regimens achieved lower globe salvage rates than standard regimens, for both more advanced (R-E group V) and less advanced (R-E groups I–IV) eyes (Figure 3). Additionally, “enhanced” regimens, which add a fourth drug, extra cycles, or second-line agents, increase globe salvage success rates for eyes with advanced disease (Figure 4). Older age, disease severity, and treatment regimen are each independent predictors of poorer outcome.

### 4.2. Limitations and Risks for Bias

This meta-analysis compares data across 27 studies to assess the relative efficacy for globe salvage among standard, reduced-intensity, and enhanced-intensity IV CRD regimens. There is an inherent risk of publication bias across all studies included in this meta-analysis. In addition, within studies, there may be treatment bias, as patients with more severe disease are sometimes offered enucleation primarily, rather than attempting globe salvage with CRD. In the majority of publications, the authors only reported on those patients in whom globe salvage was attempted with IV CRD. This is especially true for more advanced disease groups, such as ICRB group D and E eyes (since globe salvage is attempted in nearly all RE group A, B, and C eyes). This translates to a risk of bias across all studies in that the true rate of salvage with IV CRD for all eyes presenting with group D or E disease has an inherent risk for being overestimated.

As with all meta-analyses, a main limitation is the lack of standardization across studies, with variation in patient inclusion criteria and treatment strategies. Most studies did not include eyes that were enucleated primarily. It is well known that there is significant variation between treatment centers in aggressiveness in attempting to salvage eyes with more advanced disease. For example, a recent international survey showed rates of attempted globe salvage for Group D retinoblastoma range from 0% (all eyes enucleated primarily) to 100% (globe salvage with conservative therapies attempted in all cases) [55]. There is significant variation of severity even within a single classification group–in other words, not all group D eyes are equal. Thus, globe salvage success rates are overestimated, and the degree of over-estimation increases, as the severity of disease and eye classification increase. In particular, since the vast majority of group E eyes have historically undergone primary enucleation, the reported success rate for group E eyes represents only those eyes that were deemed to have potentially treatable disease, and thus represent the mildest cases that still qualified for group E classification. Therefore, the majority of studies included in this meta-analysis suffer from both selection and treatment biases when it comes to reporting treatment successes, especially for more advanced (R–E group V and ICRB group D and E) eyes.

There are a large number of variables in the treatment of retinoblastoma, and these were not consistent among the various studies included in this meta-analysis. For example, centers vary in when focal therapies are applied (started immediately vs. after 2–3 cycles of chemotherapy vs. after completion of chemoreduction), as well as the specific ways in which the therapies are applied. These were not always specified in each paper, and even if they were, there would be almost as many variations on treatment strategies as there were papers. Therefore, it was necessary to combine these all under the rubric of “consolidative therapy” for this meta-analysis. This is in no way to minimize the importance of decisions regarding how and when to apply consolidation, but rather a practical consideration for analysis purposes, especially for a rare disease like RB.

Similarly, it was necessary to accept the eye group classifications and treatment decisions that had been made by the treating physicians at the time. Inclusion criteria were as allowed by the individual studies, including any prior treatments. Length of follow-up was as reported in the individual studies, and we do not know if reportedly “successfully treated” eyes subsequently had late recurrences after the original study paper was published (although all studies did not include patients who were still undergoing therapy actively at the time of publication). Similarly, we had to accept the authors’ determinations regarding treatment success, or treatment failure that necessitated enucleation. A related form of bias in all such studies is that treating physicians might be more inclined to continue aggressive treatment and avoid enucleation in bilaterally affected children, vs. in unilaterally affected patients where the second unaffected eye continues to provide useful vision. A similar problem arises in determining the success of IVC now that IAC is readily available for rescue. Since the majority of studies analyzed in this meta-analysis were conducted before the widespread adoptions of IAC (or intravitreal chemotherapy) in most centers, this was less of an issue in conducting this meta-analysis than it might be in clinical decision making going forward.

Of all the studies with full data available to review, there were only three studies that included information on eyes that were primarily enucleated. It can be reasonably assumed that primarily enucleated eyes were more severe and would have been more likely to have failed treatment if conservative globe salvage CRD had been attempted. However, given that the overwhelming majority of studies did not report data on primarily enucleated eyes, we elected not to include primary enucleations (reported in only 3 studies) as treatment failures, but rather removed these primarily enucleated eyes from consideration for this small number of studies. We acknowledge that as a result, we may be overestimating the globe salvage rate for the most advanced eyes. However, the analysis was performed in this way, in order to maintain consistency among all reports and to avoid penalizing those studies that chose to attempt globe salvage CRD in the most advanced eyes or which were forthright in including data on primarily enucleated eyes. Our analysis therefore attempts to provide a best approximation of the likelihood of globe salvage, based on the best data available, for the treatment regimen that was actually used.

This meta-analysis is also limited by the longstanding issue of the use of different retinoblastoma classification systems across the reported literature. While R-E and ICRB/IIRC vary in their classification criteria, this meta-analysis investigated the data that were reported based on the classification systems the published studies used (ICRB/IIRC). No papers reported on the American Joint Committee on Cancer (AJCC) classification system, as it was not in widespread use at the time of publication of many of these studies. Studies published after 1st January 2017 were purposefully excluded in order to exclude data that may be confounded by other treatments such as intravitreal chemotherapies, rather than IV CRD alone. As it stands, the AJCC was not used to classify RB at that time and it is still not the most commonly used classification for RB today.

There are also subtle differences (most notably in the classification of group E eyes) in the two published versions of the “International Classification” (the International Classification of Retinoblastoma [ICRB]/Philadelphia system, and the International Intraocular Classification System [IIRC]/Children’s Hospital of Los Angeles [CHLA/Murphree] system). This is a longstanding and well-known problem with the RB literature in general, as it relates to reporting of results. While it would be ideal to separate out the Philadelphia vs. CHLA versions for a meta-analysis such as this, there simply are not enough published studies to separate out the two systems while allowing for meaningful data analysis. Therefore, we have grouped these two systems together for this analysis. Furthermore, the discrepancies do not affect the R-E classified data, nor does it affect the data for ICRB Groups A, B and C eyes. Only Groups D and E eyes are affected.

Novetsky et al. 2009 demonstrated that reclassification between the two systems only affected group assignment of 5.2% of eyes and primarily affected group E eyes [56]. Furthermore, the eyes in question (very bad Group D eyes, or group E eyes) are the eyes that are less likely to be treated conservatively with IV CRD, as these are the most difficult to treat with globe salvaging therapy and are the most likely eyes to be primarily enucleated, regardless of how a particular study chooses to classify the eyes (Philadelphia vs. CHLA). Only a very small percentage of group E eyes are likely to be selected for attempted IV CRD over enucleation. Therefore, regardless of whether an eye is classified as a very advanced D eye or as an E eye, it is somewhat less likely to undergo treatment over enucleation. Therefore, although there exist subtle classification differences of eyes between the two ICRB systems, this limitation would affect only a small percentage of advanced eyes (Ds and Es) and only a small percentage of these eyes would be considered for treatment with IV CRD over enucleation. Stated differently, the small number of eyes that are reclassified between E and D based on which version of the International System is used is far outweighed by the impact that treatment bias and selection bias would have on these eyes, regardless of how they are classified. Therefore, the impact of this limitation on this meta-analysis is likely relatively small.

Those studies that reported individual patient-level data were more likely to be those studies with smaller numbers of patients, as reporting patient-level data on very large numbers of patients is often not feasible in published manuscripts. However, one may assume that larger studies come from hospitals with the greatest treatment experience. Thus, the exclusion of these larger centers, which did not provide patient level data, from the patient level analysis may be a potential source for selection bias.

We defined treatment failure as those eyes requiring enucleation or requiring salvage EBRT. The need for salvage EBRT was included as treatment failure because the CRD being evaluated had been unable to salvage the eye in question, thus necessitating radiotherapy. In addition, the decision to treat with EBRT carries a risk of iatrogenic second malignant neoplasms among patients with a germline RB1 mutation, and so many centers choose enucleation over salvage radiotherapy. A small number of studies had to be excluded because it was not possible to differentiate eyes that had received salvage EBRT, eyes that had been secondarily enucleated, and eyes that were treated with salvage EBRT then subsequently enucleated. Were these few studies included, “failures” that received both radiotherapy and subsequent enucleation might have been double-counted.

One point to ponder is why the handful of ICRB group A and Reese–Ellsworth group I eyes were treated with IVC, when presumably these eyes with very limited disease would have been amenable to upfront local therapies such as laser photocoagulation or cryotherapy for definitive management. While we cannot answer these questions definitively on behalf of the authors of the original papers, there are two likely scenarios: (1) These eyes may have represented contralateral eyes of bilaterally affected patients with more advanced disease in the other eye that necessitated systemic chemoreduction. (2) These were bilaterally affected patients in whom the other (advanced) eye required enucleation and was found to have pathologic high risk features necessitating chemotherapy for adjuvant treatment of presumed micrometastatic disease. In both these scenarios, we assume that the treating physician might have opted to allow the chemotherapy (which would be required anyway) to treat the remaining group A (or group I) eye before applying consolidative treatments.

Lastly, this meta-analysis focused on the endpoint of globe salvage, as defined in the Methods section. There is relatively scant literature regarding visual outcomes of patients treated for RB [57]. In the future, this will be an increasingly important endpoint to assess, particularly in decision-making regarding IVC vs. IAC. The RIVERBOAT Consortium (Research Into Visual Endpoints and RB health Outcomes After Treatment; NCI R01CA225005), is an ongoing, NIH-funded, international, multi-center prospective study that our group is leading. The RIVERBOAT study aims to compare visual and other health-related outcomes for RB patients undergoing various treatment modalities to directly answer these important questions about vision.

## 5. Conclusions

### 5.1. General Conclusions

This meta-analysis:

1. Establishes a consolidated and evidence-based baseline for globe salvage success rates for each retinoblastoma classification for various intravenous chemoreduction regimens, against which to compare new, emerging, and future therapeutic options.
2. Confirms that both the Reese–Ellsworth and International Classification of Retinoblastoma classification schemes are predictive of intravenous chemoreduction success.
3. Establishes that older age reduces the chance for successful globe salvage, regardless of disease severity and regardless of chemotherapy regimen.
4. Demonstrates that globe salvage success is affected by chemotherapy regimen. For both less severely affected eyes as well as for eyes with advanced disease, globe salvage rates were lower for two-drug regimens. For more severely affected eyes with vitreous seeds, enhanced regimens have higher success rates than standard intravenous chemotherapy, and thus the use of a higher-intensity regimen might be considered in advanced eyes (that are not candidates for IAC).
5. The published retinoblastoma literature lacks standardized reporting and is fraught with treatment bias and selection bias. The field would benefit from agreed-upon standard reporting of patient inclusion and outcomes.

### 5.2. Importance of This Work

Given the increasing shift toward replacing standard intravenous chemoreduction with intra-arterial chemotherapy and incorporating adjuvant intravitreal chemotherapy, this meta-analysis consolidates and analyzes the published results of various intravenous chemoreduction regimens. These results are timely and important so that we can scientifically compare whether (and by how much) novel treatments such as intra-arterial or intravitreal chemotherapy improve patient-based outcomes. For physicians employing intravenous chemoreduction, this meta-analysis provides evidence-based guidance in selecting an intravenous chemotherapy regimen based on the disease severity of their patients. This can help guide future directions of outcomes-based research.

## Figures and Tables

**Table 1:** Characteristics of studies included in meta-analysis.

Author(s)	Year	Regimen	Total Eyes	Patient Level Data	Reese-Ellsworth Classification (R-E) Number of Eyes					International Classification of RB (ICRB)/International Intraocular RB Classification (IIRC) Number of Eyes				
					I	II	III	IV	V	A	B	C	D	E
Brichard et al	2002 [33]	Standard /Reduced	24	Yes	4	3	5	0	12	-	-	-	-	-
Hee Yoo et al	2002 [44]	Standard /Enhanced	10	Yes	0	0	0	2	8	-	-	-	-	-
Kim et al	2003 [32]	Standard /Enhanced	27	Yes	2	7	5	7	6	-	-	-	-	-
Menon et al	2007 [45]	Standard /Enhanced	25	Yes	1	5	11	7	1	-	-	-	-	-
Bartuma et al	2014 [46]	Standard	46	Yes	-	-	-	-	-	8	25	1	11	1
Greenwald et al	1996 [34]	Reduced	11	Yes	0	1	3	1	6	-	-	-	-	-
Levy et al	1998 [40]	Reduced	38	Yes	2	1	12	3	20	-	-	-	-	-
Beck et al	2000 [41]	Reduced	33	Yes	5	10	3	1	14	-	-	-	-	-
Wilson et al	2001 [42]	Reduced	34	Yes	5	11	2	2	14	-	-	-	-	-
Rodriguez-Galindo et al	2003 [35]	Reduced	43	Yes	7	12	5	3	16	-	-	-	-	-
Wilson et al	2005 [36]	Reduced	27	Yes	4	1	2	0	20	-	-	-	-	-
Dunkel et al	2007 [37]	Reduced	43	Yes	13	5	13	2	10	-	-	-	-	-
Gallie et al	1996 [4]	Enhanced	38	Yes ^a^	9	7	4	2	16	-	-	-	-	-
Manjandavida et al	2014 [43]	Enhanced	101	Yes ^a^	0	0	0	0	101	0	0	21	40	40
Murphree et al	1996 [5]	Standard /Reduced	73	No	18	16	4	4	31	-	-	-	-	-
Gündüz et al	1998 [39]	Standard	27	No	0	0	0	0	27	-	-	-	-	-
Antoneli et al	2006 [29]	Standard	145	No	22	16	13	11	83	-	-	-	-	-
Shields et al	2006 [52]	Standard	249	No	27	53	78	37	54	23	96	21	109	0
Schefler et al	2007 [53]	Standard	44	No	1	6	3	5	29	-	-	-	-	-
Cohen et al	2009 [50]	Standard	18	No	-	-	-	-	-	0	0	0	18	0
Berry et al	2013 [51]	Standard	55	No	-	-	-	-	-	0	0	0	55	0
Schiavetti et al	2005 [30]	Reduced	58	No	10	16	9	6	17	-	-	-	-	-
Zage et al	2008 [49]	Reduced	48	No	6	7	9	1	25	7	15	8	18	0
Lumbroso-Le Rouic et al	2016 [38]	Reduced	65	No	11	15	25	5	9	3	32	11	19	0
Chung et al	2008 [47]	Enhanced	80	No	4	7	8	8	53	6	19	6	30	0
Young Shin et al	2010 [48]	Enhanced	65	No	7	15	18	15	10	8	14	0	42	1
Künkele et al	2013 [31]	Enhanced	56	No	-	-	-	-	-	4	40	6	6	0

^a^ Individual patient level data were included in this study, but covariates such as patient age were not provided.
